# Fabrication of Sn@Al_2_O_3_ Core-shell Nanoparticles for Stable Nonvolatile Memory Applications

**DOI:** 10.3390/ma12193111

**Published:** 2019-09-24

**Authors:** Jong-Hwan Yoon

**Affiliations:** Department of Physics, College of Natural Sciences, Kangwon National University, 1 Kangwondaehak-gil, Chuncheon, Gangwon-do 24341, Korea; jhyoon@kangwon.ac.kr; Tel.: +82-10-6393-8466

**Keywords:** core-shell nanoparticle, stacked tunneling layer, nonvolatile memory, charge retention

## Abstract

Sn@Al_2_O_3_ core-shell nanoparticles (NPs) with narrow spatial distributions were synthesized in silicon dioxide (SiO_2_). These Sn@Al_2_O_3_ core-shell NPs were self-assembled by thermally annealing a stacked structure of SiO_x_/Al/Sn/Al/SiO_x_ sandwiched between two SiO_2_ layers at low temperatures. The resultant structure provided a well-defined Sn NP floating gate with a SiO_2_/Al_2_O_3_ dielectric stacked tunneling barrier. Capacitance-voltage (C-V) measurements on a metal-oxide-semiconductor (MOS) capacitor with a Sn@Al_2_O_3_ core-shell NP floating gate confirmed an ultra-high charge storage stability, and the multiple trapping of electron at the NPs, as expected from low-*k*/high-*k* dielectric stacked tunneling layers and metallic NPs, respectively.

## 1. Introduction

In recent years, the demand for smaller nonvolatile memory (NVM) devices with higher operating speeds, larger storage capacities, and higher data storage stabilities has significantly increased. When designing and preparing such NVM devices, three important factors should be considered. Firstly, a floating gate consisting of discrete charge traps (nanocrystals (NCs)) [1,2] can be used, where charges trapped at discrete sites are more stable, due to no or little charge loss taking place at a floating gate through a single leakage path in the tunneling oxide. This allows the scaling of devices to smaller dimensions by reducing the tunneling oxide thickness. Secondly, metallic NCs can be employed as discrete charge sites [3,4,5], which reduce the spurious effects caused by traps at the NCs/oxide interface, and enhance the charge storage capacity due to multiple electron trapping at the NCs [3]. Thirdly, a low-*k*/high-*k* dielectric stacked tunneling oxide barrier can be employed instead of a single dielectric layer of SiO_2_ [6,7,8], with one example including SiO_2_/Al_2_O_3_, which produces a device exhibiting a high charge storage stability. These three factors can be achieved in a facile manner by forming metal@high-*k* dielectric core-shell nanoparticles (NPs) between tunneling and control oxide layers. Recently, we observed that an Al_2_O_3_ high-*k* dielectric can be easily formed by the thermal annealing of a SiO_x_/Al bilayer at 550 °C [9]. This observation was employed to form metal/Al_2_O_3_ core-shell NPs in SiO_2_, which results in the metal NC floating gate with a low-*k*/high-*k* dielectric stacked tunneling layer. 

Thus, we herein report the self-assembly of Sn@Al_2_O_3_ core-shell NPs between tunneling, and control SiO_2_ layers by the thermal annealing of a stack of SiO_x_/Al/Sn/Al/SiO_x_ sandwiched between two SiO_2_ layers at low temperatures below 550 °C. This method should allow a facile resolution of the three key points mentioned above to improve the performances of NVM devices. In particular, we expect that the low-temperature fabrication technique described herein is a promising alternative to produce future NVM devices, including flexible memory devices. 

## 2. Materials and Methods 

**Figure 1:**
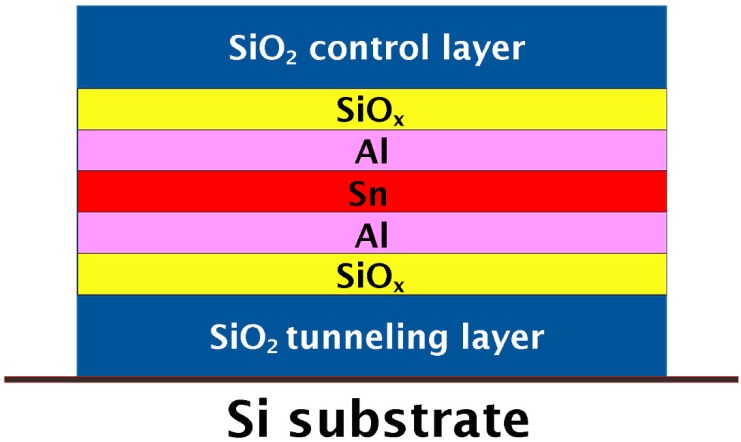
Cross-sectional schematic representation of the stacked structure employed in preparation of the Sn@Al_2_O_3_ core-shell nanoparticles.

Sn@Al_2_O_3_ core-shell NPs were formed in SiO_2_ by the thermal annealing of a stacked structure of SiO_x_/Al/Sn/Al/SiO_x_ sandwiched between two SiO_2_ layers. The SiO_x_ layers were used for the facile formation of the Al_2_O_3_ phase. The required stacked structures for core-shell NP formation were produced as follows. A thin SiO_2_ layer, which acts as a tunneling barrier, was initially grown on the Si substrate, and a thin SiO_x_ layer was deposited on the obtained SiO_2_ layer. Subsequently, a thin Al layer, a thin Sn layer, and a thin Al layer were deposited using a conventional thermal evaporation system. Finally, a thin SiO_x_ layer was grown on the Al/Sn/Al layered structure, followed by the growth of a thick SiO_2_ layer which acts as a control gate oxide. The SiO_x_ and SiO_2_ layers were deposited at a substrate temperature of 300 °C by plasma-enhanced chemical vapor deposition using different fixed flow rates of SiH_4_ and N_2_O. The chemical composition of the SiO_x_ films were analyzed using energy recoil detection (ERD) spectroscopy. A cross-sectional schematic representation of the stacked structure is shown in Figure 1.

The Sn@Al_2_O_3_ core-shell NPs were formed by thermal annealing of the prepared sandwich structure at low temperatures ≤550 °C in a quartz-tube furnace with high-purity (99.999%) nitrogen gas at a flow rate of 20 mL/min. The annealing N_2_ gas was dried prior to entering the furnace by passing it through a drying canister filled with anhydrous CaSO_4_. 

The morphology and microstructure of the core-shell NPs were analyzed by transmission electron microscopy (TEM) using a JEOL JEM-2100F (Jeol, Tokyo, Japan) instrument operating at 200 kV. The chemical compositions of the NPs were analyzed by energy dispersive X-ray spectroscopy (EDX) using an Aztec E-Max^N^ (Oxford, High Wycombe, UK) attached to the TEM instrument. 

The memory properties of the core-shell NPs were investigated by measuring the Capacitance-voltage (C-V) characteristics using a metal-oxide-semiconductor (MOS) capacitor with floating gates based on Sn@Al_2_O_3_ core-shell NPs. The MOS capacitors were prepared by evaporating Al metal onto a control oxide layer through a mask containing circular holes with an area of 0.09 mm^2^. C-V measurements were carried out using a Keithley 590 CV analyzer (Keithley, Ohio, USA) with a high frequency of 1 MHz at room temperature (~25 °C). The programming and erasing operations were carried out using a Keithley 230 programmable voltage source (Keithley, Ohio, USA).

## 3. Results and Discussion

**Figure 2:**
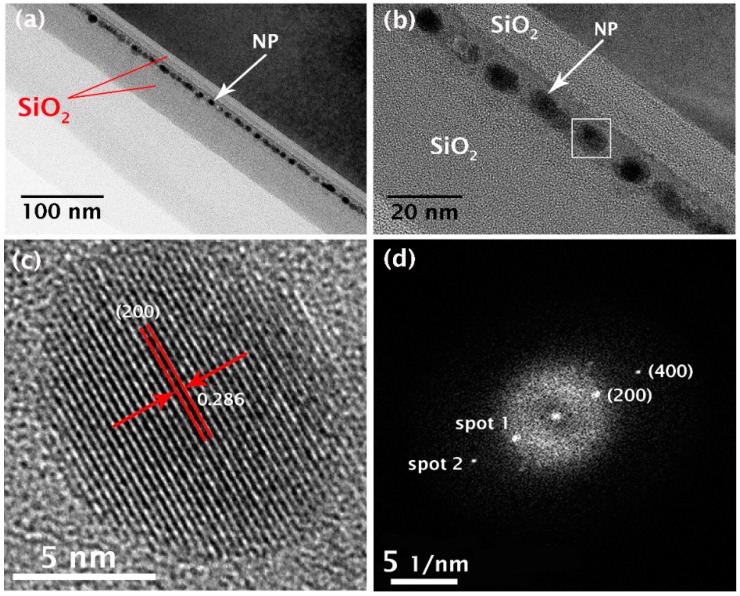
TEM images of the stacked SiO_1.3_/Al/Sn/Al/SiO_1.3_ structure sandwiched between two SiO_2_ layers after annealing at 500 °C for 30 min. (**a**) Low- and (**b**) high-magnification images; (**c**) high-resolution TEM image of the nanoparticle (NP) indicated by the white square in (**b**); and (**d**) fast Fourier transform (FFT) pattern associated with the lattice structure in (**c**).

Core-shell NPs are conventionally synthesized by coating the particle (core) with a potential material (shell) to impart the desired functionality [10]. As an alternative approach, we synthesized core-shell NPs by annealing a stacked structure consisting of two materials corresponding to the core and the shell, with the present Sn@Al_2_O_3_ core-shell NPs being synthesized using a stack of Sn and Al layers as the core and the shell materials, respectively. Figure 2 shows the morphology and microstructure of the layered SiO_1.3_/Al/Sn/Al/SiO_1.3_ structure sandwiched between two SiO_2_ layers (tunneling and control layers) after annealing at 500 °C for 30 min. The thicknesses of the SiO_1.3_, Al, and Sn layers are 7, 3, and 2 nm, respectively, while the thicknesses of tunneling and control SiO_2_ layers are ~9 and ~35 nm, respectively. Figure 2a shows the overall cross-sectional transmission electron microscope (XTEM) image, and its corresponding magnification is shown in Figure 2b, where the white square represents the NP selected for detailed microstructural analysis. The images clearly show the presence of dark NPs, which are approximately spherical in shape and almost uniform in size, which are distributed from 7.4 to 8.9 nm in diameter. The average diameter is approximately 8.3 nm. Moreover, it is evident that the spatial distribution of the NPs is also narrow, thereby demonstrating the efficacy of the technique for preparing NC floating gate structures for NVM devices. Indeed, a well-defined NP monolayer with a narrow spatial distribution is essential for the fabrication of such floating gate devices.

To identify the phase of the NPs, high-resolution TEM (HRTEM) and electron diffraction (ED) measurements were performed on individual NPs. Figure 2c,d shows a representative HRTEM image and its associated fast Fourier transform (FFT) pattern, respectively, which were obtained from the NP indicated by the white square. The HRTEM image clearly shows that the NP has a well-defined boundary and a regular lattice structure consistent with it being a single crystal phase. In addition, the lattice structure reveals an inter-planar lattice spacing of 0.286 nm, as labelled in the image. This value agrees well with the inter-planar distance between the (200) planes of a β-Sn crystal. Furthermore, the FFT image shows bright diffraction spots due to single crystal lattice planes. Using the relationship, *r*_2_/*r*_1_ = (*h*_2_^2^ + *k*_2_^2^ + *l*_2_^2^)/(*h*_1_^2^ + *k*_1_^2^ + *l*_1_^2^ ), where *r*_1_ and *r*_2_ are the distances from the center (C) to spots 1 and 2, respectively, and (*h*_1,_ *k*_1_, *l*_1_) and (*h*_2_, *k*_2_, *l*_2_) are the Miler indexes of the crystal planes corresponding to spots 1 and 2, respectively, indexing of the crystallographic plane corresponding to each spot in the FFT pattern showed that spots 1 and 2 are consistent with the (200) and (400) crystal planes in the Sn crystal. These results unambiguously demonstrate that the self-assembled NPs are composed of the β-Sn phase of a single-crystal structure with preferential [100] orientation.

**Figure 3:**
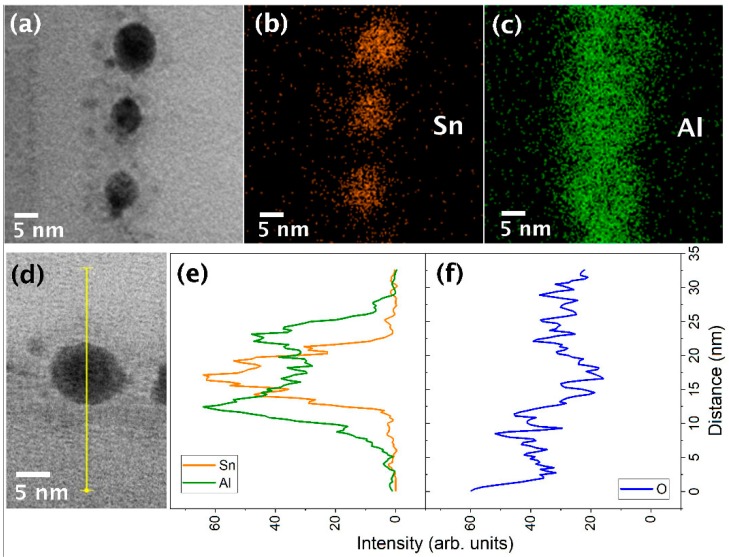
(**a**) TEM image used for the EDX mapping scans; (**b**,**c**) mapping images of Sn (yellow line) and Al (green line), respectively; (**d**) TEM image and guide line for the EDX line scans; (**e**) EDX line scanning spectra of Sn and Al; and (**f**) EDX line scanning spectrum of O.

To identify the exact phase around the NPs, EDX analyses were performed on individual NPs. Thus, Figure 3b,c show representative EDX mapping images of the Sn and Al present in the NPs shown in the TEM image of Figure 3a, respectively. These mapping images clearly show that the two elements are distributed in a nearly circular shape with a co-center, where the distribution radius of Sn is smaller than that of Al. These mapping data demonstrate that Sn is located in the inner region (core) of the NPs while Al is located on the outer region (shell) of the Sn core, supporting the premise that the prepared NPs are composed of a core-shell structure. This conclusion is further confirmed by the EDX line scans of Sn and Al. 

More specifically, Figure 3e shows representative EDX line scanning spectra of Sn and Al, which were obtained from line scans along the yellow line shown in the TEM image (Figure 3d). As indicated, the spectrum of Sn exhibits a symmetric shape with a peak at the center of the NP, which is consistent with the spectrum expected for a solid sphere composed of Sn atoms alone. In the case of Al, the EDX intensity is higher in the outer regions of the NP and presents a saddle-shaped symmetry. This feature is also in good agreement with that expected for a spherical shell. These line scanning data therefore support our above conclusion that the NPs are composed of a core and a shell, of which the main chemical components are Sn and Al, respectively.

To better understand the exact chemical composition of the Al-containing shell, EDX line scanning of O was performed. As shown in Figure 3f, a saddle-shape spectrum was observed similar to that observed in the Al spectrum, indicating that oxygen atoms are also present in the shell. This in turn suggests that the shell contains a composite phase composed of Al and O. The image shows that the thickness of the shell is approximately 7 nm. It should be noted that Al is generally known to dissociate SiO_2_ since the Al–O bond is stronger than the Si–O bond [11]. In particular, off-stoichiometric silicon oxide, SiO_x_, contains silicon oxide phases whose Si binding energy is lower than that of SiO_2_ (103.3 eV) [12,13], and so Al–O bonds can be more easily generated in the Al/SiO_x_ system than in the Al/SiO_2_ system. In addition, previous studies [9,14,15] have reported that Al_2_O_3_ is the main product of the chemical reaction between Al and SiO_x_, and so based on the above facts, it was apparent that the shell was composed mainly of Al_2_O_3_, thereby giving a Sn@Al_2_O_3_ core-shell nanostructure for the self-assembled NPs. In addition, this mechanism acts as a constraint to ensure that the Sn@Al_2_O_3_ core-shell NPs are formed between two SiO_2_ layers, and this was confirmed by the formation of the well-defined NP monolayer as shown in Figure 1.

**Figure 4:**
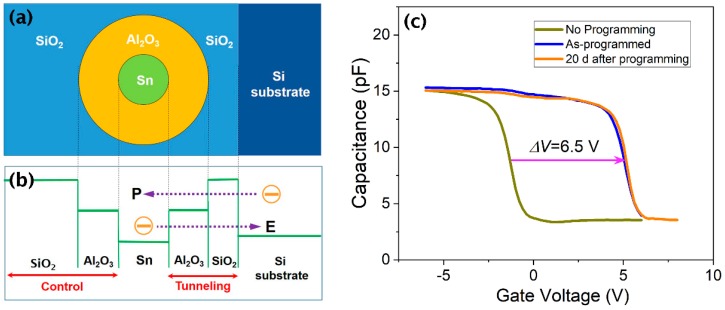
(**a**) Cross-sectional schematic representation of an Sn@Al_2_O_3_ core-shell nanoparticle embedded between tunneling and control SiO_2_ layers; (**b**) the band diagram associated with the core-shell structure shown in (**a**); and (**c**) Capacitance-voltage (C-V) curves over time after charging a Sn@Al_2_O_3_ core-shell NP floating gate. No shift in the C-V curve is observed 20 d after charging.

The preparation of Sn@Al_2_O_3_ core-shell NPs between two SiO_2_ layers results in the formation of a metal (Sn) NC floating gate with a low-*k* (SiO_2)_ and high-*k* (Al_2_O_3_) dielectric stacked tunneling barrier. Thus, Figure 4a,b shows schematic representations of the Sn@Al_2_O_3_ core-shell NP sandwiched between tunneling, and control SiO_2_ layers along with the related band diagram. This structure can potentially improve the performance of the NVM devices, and so we were interested in examining the memory characteristics, especially the charge storage stability, of a Sn@Al_2_O_3_ core-shell NP floating gate using an MOS capacitor structure. Figure 4c shows the C-V curves measured before programming (dark yellow line), immediately after programming (blue line), and 20 d after programming (yellow line). Here, a high programming gate voltage was used to store as much charge as possible in the floating gate in order to more accurately check the charge storage stability. The programming was achieved by applying a gate voltage of +30 V for 1 s, which caused a large shift of the C-V curve by ~6.5 V.

From the C-V data, we initially estimated the number (*n*) of electrons stored on a single Sn NC using the simple formula $\Delta V=\frac{d}{\varepsilon_{\mathrm{os}}}Q_{t}$, where ΔV is the voltage shift of the C-V curve upon application of a charge *Q*_t_, which in turn is the total charge stored in the NP floating gate corresponding to the area of the gate electrode (*A*). In addition, *d* is the distance between the NC floating gate and the gate electrode, and *ε*_os_ is the dielectric permittivity of SiO_2_ [16]. Using ΔV = 6.5 V, *A* = 0.09 mm^2^, *d* = 35 nm, and an areal density of Sn NCs, 1.5 × 10^12^ cm^−2^, which was estimated using the TEM image shown in Figure 2b, the value of *n* was found to be 2.6 × 10^3^. This value indicates that a large number of electrons were trapped on a single NC, a result expected from metal NCs [3]. In particular, the high multiple trapping of electrons renders it possible to realize multibit memory [17,18] for enhancement of data storage capacity.

Thus, the charge storage stability of the presented Sn@Al_2_O_3_ core-shell NP floating gate was explored by monitoring the shift of the C-V curve over time after electron charging on the floating gate. The C-V curve shifts in proportion to the amount of charge stored in the floating gate, and so loss of the charge tapped in the floating gate causes a shift in the C-V curve. In the present case, no shift was observed for the C-V curve even 20 d after an electron charging of 2.6 × 10^3^ per NC (Figure 4c). This lack of shift in the C-V curve indicates that there is no loss of charge trapped in the floating gate, which in turn implies a significantly more stable charge storage. When no erasing voltage is applied, the electrons trapped in the floating gate can be de-trapped into Si by tunneling emission through the tunneling barrier (as indicated by the dotted line E). In the present case, the SiO_2_/Al_2_O_3_ stacked layer acts as a tunneling barrier, where the equivalent oxide thickness [19] of the Al_2_O_3_ layer was added to that of the SiO_2_ layer. This increase in the thickness of the tunneling gate oxide layer results in a reduction in the electron tunneling emission rate as compared to the case of a single SiO_2_ tunneling layer, and so significantly increases the charge retention time, i.e., the charge storage stability. On the other hand, the other memory characteristics, such as programming speed (as indicated by the dotted line P) [6,7], strongly depend on the thickness of the low-*k* dielectric (SiO_2_) layer. Therefore, these memory characteristics have not been tested here, since it would be worthwhile to examine after optimizing the thickness of the SiO_2_ layer. The full memory characteristics of this structure will be examined separately in future studies.

## 4. Conclusions

In conclusion, we successfully developed a simple method to fabricate a Sn nanocrystal floating gate with a SiO_2_/Al_2_O_3_ dielectric stacked tunneling barrier that is suitable for highly functional NVM devices. This memory structure was easily formed through the preparation of Sn@Al_2_O_3_ core-shell NPs between tunneling and control SiO_2_ layers, where the formation of the Sn@Al_2_O_3_ core-shell NPs themselves was achieved by thermal annealing of a stack of SiO_1.3_/Al/Sn/Al/SiO_1.3_ sandwiched between tunneling and control SiO_2_ layers at low temperatures. Importantly, capacitance-voltage (C-V) measurements on a metal-oxide-semiconductor capacitor with a Sn@Al_2_O_3_ core-shell NP floating gate confirmed an ultra-high charge storage stability, as expected from low-*k*/high-*k* dielectric stacked tunneling barrier. This study is of relevance as the resulting Sn@Al_2_O_3_ core-shell NPs were demonstrated to form a well-defined metallic NC floating gate with a stacked dielectric tunneling layer, and excellent memory characteristics suitable for stable NVM device applications.
